# Relationship between Depression and Anxiety during Pregnancy, Delivery-Related Outcomes, and Healthcare Utilization in Michigan Medicaid, 2012–2021

**DOI:** 10.3390/healthcare11222921

**Published:** 2023-11-07

**Authors:** Kara Zivin, Xiaosong Zhang, Anca Tilea, Sarah J. Clark, Stephanie V. Hall

**Affiliations:** 1 Center for Clinical Management Research, VA Ann Arbor Healthcare System, Ann Arbor, MI 48105, USA; 2 Department of Psychiatry, Michigan Medicine, Ann Arbor, MI 48109, USA; 3 Department of Obstetrics and Gynecology, Michigan Medicine, Ann Arbor, MI 48109, USA; 4 Ambulatory Care Program, Department of Pediatrics, Michigan Medicine, Ann Arbor, MI 48109, USA; 5 Department of Learning Health Sciences, Michigan Medicine, Ann Arbor, MI 48109, USA

**Keywords:** pregnancy, depression, anxiety, maternal health, infant health, maternal health services, medicaid

## Abstract

To evaluate associations between depression and/or anxiety disorders during pregnancy (DAP), delivery-related outcomes, and healthcare utilization among individuals with Michigan Medicaid-funded deliveries. We conducted a retrospective delivery-level analysis comparing delivery-related outcomes and healthcare utilization among individuals with and without DAP between January 2012 and September 2021. We used generalized estimating equation models assessing cesarean and preterm delivery; 30-day readmission after delivery; severe maternal morbidity within 42 days of delivery; and ambulatory, inpatient, emergency department or observation (ED), psychotherapy, or substance use disorders (SUD) visits during pregnancy. We adjusted models for age, race/ethnicity, urbanicity, federal poverty level, and obstetric comorbidities. Among 170,002 Michigan Medicaid enrollees with 218,890 deliveries, 29,665 (13.6%) had diagnoses of DAP. Compared to those without DAP, individuals with DAP were more often White, rural dwelling, had lower income, and had more comorbidities. In adjusted models, deliveries with DAP had higher odds of cesarean and preterm delivery OR = 1.02, 95% CI: [1.00, 1.05] and OR = 1.15, 95% CI: [1.11, 1.19] respectively), readmission within 30 days postpartum (OR = 1.14, 95% CI: [1.07, 1.22]), SMM within 42 days (OR = 1.27, 95% CI: [1.18, 1.38]), and utilization compared to those without DAP diagnoses (ambulatory: OR = 7.75, 95% CI: [6.75, 8.88], inpatient: OR = 1.13, 95% CI: [1.11, 1.15], ED: OR = 1.86, 95% CI: [1.80, 1.92], psychotherapy: OR = 172.8, 95% CI: [160.10, 186.58], and SUD: OR = 5.6, 95% CI: [5.37, 5.85]). Among delivering individuals in Michigan Medicaid, DAP had significant associations with adverse delivery-related outcomes and greater healthcare use. Early detection and intervention to address mental illness during pregnancy may help mitigate burdens of these complex yet treatable disorders.

## 1. Introduction

Pregnant individuals with mental health conditions deliver more than 800,000 infants each year [1]. Up to or exceeding 20% of individuals have depression and/or anxiety during pregnancy (DAP) [2,3,4,5,6], the most underdiagnosed obstetric complication in the United States (US) [7]. Individuals with lower incomes suffer at a higher rate (40–60%) and experience more severe episodes of DAP [7]. The estimated societal financial toll of not treating DAP exceeds $14 billion annually in the US, with healthcare expenditures and cesarean deliveries driving the overall economic burden [8].

The US experiences the worst maternal morbidity and mortality rates of any peer nation [9]. The global maternal mortality rate decreased 43% since 1990, while the US maternal mortality rate increased 16% from 12 to 14 deaths per 100,000 live births [2]. The US maternal mortality rate remains 75% higher than the average maternal mortality rate for high income nations [3]. High cesarean and preterm delivery rates drive severe maternal morbidity (SMM) and maternal death [4], often along socioeconomic and racial lines [10,11]. Disparities further influence utilization patterns as high-risk individuals seek emergency services to supplement inadequate or inaccessible perinatal health care [5].

Literature indicates that maternal depression and anxiety may increase odds of preterm birth, low birthweight, and other poor birth outcomes [6,7]; however, some systematic reviews maintain that the association is inconclusive [8] or reflects complex causal pathways [7]. A recent study using nationally representative survey data found that individuals with depressive symptoms during pregnancy had higher odds of going without routine medical care and using urgent care more frequently compared to delivering individuals without these symptoms [9]. This study will complement and extend that work by comparing associations between individuals with and without diagnosed DAP and the amounts and types of health care utilization in a high-need population of delivering individuals, namely, those with Medicaid health insurance coverage, which funds nearly half of all births [10,11].

We aimed to determine if delivering individuals with DAP had higher rates of suboptimal delivery-related outcomes and greater utilization of health services compared to delivering individuals without diagnosed DAP.

## 2. Materials and Methods

We conducted a retrospective cohort study to evaluate health care utilization for Michigan Medicaid enrollees aged 15 to 49 who delivered live births between 1 January 2012 and 30 September 2021 [12]. We used de-identified administrative claims from the Michigan Department of Health and Human Services (MDHHS) data warehouse. During the study period, Medicaid served as the primary payer for 40–44% of births in Michigan [13].

Our inclusion criteria required continuous enrollment in Michigan Medicaid for nine months prior to delivery and three months afterward. Although we recognize that not all pregnancies last exactly nine months, we aimed to increase the likelihood that we could observe all health services utilization during pregnancy. Similarly, Medicaid is available to an eligible woman while she is pregnant, including the month her pregnancy ends and during the two calendar months following the month her pregnancy ends (i.e., 2–3 months). Individuals could appear in our dataset more than once if they had more than one delivery during the study period.

An honest broker extracted and created a de-identified study dataset of Michigan Medicaid enrollment and administrative claims data. The eligible population included all deliveries with at least one live birth. For eligible enrollees, the dataset included all paid claims for nine months prior to and up to three months after the delivery date, including visit dates, hospital admission, and discharge dates; International Classification of Diseases, Ninth Revision, Clinical Modification (ICD-9-CM and ICD-10) codes (diagnostic and procedure codes); Diagnosis Related Group (DRG) codes; Current Procedural Terminology, 4th edition, codes (procedure codes); Healthcare Common Procedure Coding System codes (supplies and services codes); Revenue codes, Type of Bill codes, HIC3 Specific Therapeutic Codes; and prescription dispense date. Demographic data at the time of delivery included year of birth, race/ethnicity, Medicaid benefit plan, zip codes, and income level (% Federal Poverty Level [FPL]). We did not exclude individuals with third party liability, which comprised less than 3% of the study sample. Appendix A provides cohort and all variable definitions.

The University of Michigan and Michigan Department of Health and Human Services Institutional Review Boards approved this study. We followed Strengthening the Reporting of Observational Studies in Epidemiology (STROBE) reporting guidelines [14].

### 2.1. Dependent Variables

This study included four delivery-related outcomes and five utilization outcomes during the pregnancy. Delivery-related health outcomes included cesarean delivery, preterm delivery (by diagnosis of gestational age ≤37 weeks), 30-day readmission after delivery, and severe maternal morbidity (SMM) within 42 days as defined by the Centers for Disease Control and Prevention (CDC) [15]. Utilization outcomes included (1) ambulatory care visits (i.e., all outpatient visits including prenatal care); (2) inpatient visits (excluding delivery hospitalization); (3) emergency (ED) and/or observation visits; (4) psychotherapy visits; and (5) substance use disorder (SUD) treatment visits. Utilization variables include visits in the nine months before delivery.

### 2.2. Independent Variables

For this study, we considered DAP to include any diagnosis of depression or anxiety disorder during pregnancy as the primary independent variable. We included maternal sociodemographic characteristics available in the Medicaid data for time of delivery with plausible associations with outcomes of interest, including age (range 15–49); race/ethnicity (American Indian/Alaskan Native, Asian, Black, Hispanic, Native Hawaiian/Pacific Islander, White, other/unspecified); urbanicity (urban, non-urban/unknown based on zip codes of residence) [16]; and percentage of FPL (0, >0 to ≤50, >50, unknown).

We also included clinical characteristics commonly associated with outcomes of interest using any diagnosis code (not just primary diagnosis), including Bateman Obstetric Comorbidity Index (OBCMI; categorized as 0–1, 2+) [17]; behavioral health diagnoses other than depression and anxiety (serious mental illness, SUD, other behavioral health diagnoses, any of the four preceding diagnosis groups); and psychotropic medication use (antidepressants, anxiolytics, antipsychotics, mood stabilizers, opioid analgesics, medication for opioid use disorder, stimulants, any of the seven psychotropic medication classes).

### 2.3. Statistical Methods

First, we compared sociodemographic and clinical characteristics among deliveries to childbearing individuals with and without DAP at any time during the study period. Next, we used unadjusted and adjusted odds ratios from generalized estimating equation (GEE) regression in GENMOD models to assess delivery-related and utilization outcomes with delivery as the unit of analysis. We treated deliveries as a repeated measure to account for mothers who had more than one delivery during the study period. We used a compound symmetry assumption (type = cs) for the covariance matrix. We conducted all analyses using SAS 9.4 and used the PROC GENMOD module.

## 3. Results

**Figure 1:**
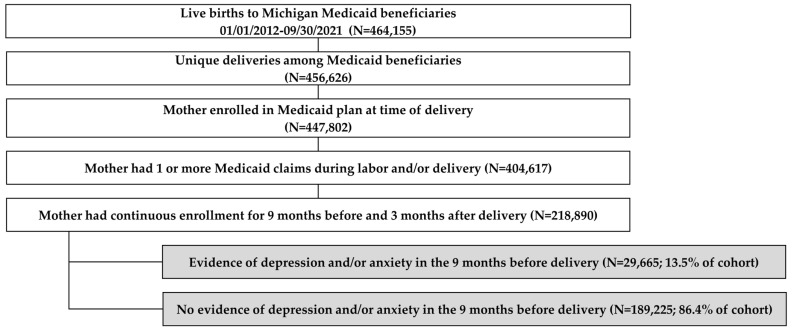
Cohort selection and attrition (unit of analysis: delivery).

The sample included 218,890 deliveries among 170,002 Michigan Medicaid enrollees meeting study criteria during the study period. This included 129,903 (76.4%) with one delivery, 32,629 (19.2%) with two deliveries, 6329 (3.7%) with three deliveries, and 1141 (0.7%) with four or more deliveries. Of those delivering individuals, 15.7% of deliveries had diagnosed DAP during pregnancy. Figure 1 presents the study cohort selection and attrition.

Table 1 compares sociodemographic characteristics and clinical characteristics of childbearing individuals with and without DAP (all *p* < 0.001). Deliveries with DAP had a higher proportion of White (69.6% versus 50.57%) enrollees than deliveries without DAP, as is consistent with other literature [18]. Deliveries with DAP also had a higher proportion of non-urban (20.3% versus 13.5%) and lower income (60.6% versus 55.8% with income at 0% FPL) enrollees than deliveries without DAP. Individuals with DAP had more obstetric comorbidities than those without DAP (2.07 vs. 1.27). Findings indicated substantially higher rates of other behavioral health diagnoses and psychotropic medication use among those with diagnosed DAP versus those without. Among deliveries with DAP, 49.6% had another behavioral health diagnosis, and 71.7% had any psychotropic medication use. Among those without diagnosed DAP, 8.8% had another behavioral health diagnosis, and 12.5% had any psychotropic medication use during pregnancy and/or postpartum.

Table 2 presents findings on delivery-related outcomes and utilization of health services from adjusted and unadjusted GEE regression models displaying odds ratios. In adjusted analyses, deliveries with DAP during pregnancy versus without DAP had 2% greater odds of cesarean delivery (OR = 1.02, [95% CI: 1.00, 1.05]) and 15% greater odds of preterm delivery (OR = 1.15, [95% CI: 1.11, 1.19]). Women with DAP had 14% greater odds of being readmitted to the hospital within 30 days of delivery (OR = 1.14, [95% CI: 1.07, 1.22]) and 27% greater odds of experiencing SMM within 42 days of delivery (OR = 1.27, [95% CI: 1.18, 1.38]).

Compared with delivering individuals without diagnosed DAP, those with pregnancy-related diagnosed DAP had a higher probability of all five types of utilization assessed during pregnancy, including an almost eight-fold increase in odds of attending an ambulatory visit (OR = 7.75, [95% CI: 6.75, 8.88]), a 13% increase in odds of attending inpatient visits (OR = 1.13, [95% CI: 1.11, 1.15]), and an 86% increase in odds of being admitted for ED/observation (OR = 1.86, [95% CI: 1.80, 1.92]). Unsurprisingly, DAP was associated with 172.8 times greater odds of attending psychotherapy visits (OR = 172.83, [95% CI: 160.10, 186.58]) and 5.61 times greater odds of attending an SUD visit (OR = 5.61, [95% CI: 5.37, 5.85]).

## 4. Discussion

This study demonstrates the magnitude of the excess healthcare use associated with DAP among delivering individuals with Medicaid. Delivering individuals with diagnosed DAP had higher rates of comorbid mental health and substance use diagnoses, higher use of psychotropic medication, higher rates of adverse delivery-related outcomes, and higher utilization than their counterparts without diagnosed DAP. Although higher rates of adverse delivery-related outcomes may be driven by associations with other characteristics besides DAP status, healthcare utilization rates remained higher for those with DAP in adjusted models, even for non-behavioral health-related services.

Although this study indicated that those with DAP had higher healthcare utilization, some of that excess utilization may represent appropriate and beneficial care, whereas other utilization may have included inappropriate or low value treatment. Individuals with DAP may need more services; however, we cannot determine from this analysis whether the care received represented the right amount and mix or type of services. Ideally, individuals with DAP would receive necessary and high value care as appropriate to their conditions; their utilization patterns would not necessarily match those for individuals without DAP.

These findings are consistent with existing literature, which consistently identifies an association between depression and/or anxiety and preterm birth [6,7,8]. However, the literature on cesarean delivery is decidedly more mixed. One systematic review found an overall null association between antenatal anxiety and cesarean delivery [6]. These null results may reflect heterogeneity within patients with DAP, as untreated DAP increased risk of cesarean delivery, while treated DAP decreased risk of cesarean delivery. Our study did not control for treatment status, but Medicaid recipients are less likely to receive postpartum mental health treatment than privately insured individuals [19], which may also occur in the antenatal population. We did not identify literature associating DAP with SMM or 30-day rehospitalization. The prevalence of both SMM and DAP has increased over the past decade [20,21], and our findings may indicate that these increases are linked.

These findings correspond with other literature on the higher healthcare utilization among those with behavioral health conditions compared to those without these conditions in delivering and non-delivering populations [9,22,23,24,25]. A five-year retrospective cohort study of Medicaid-insured individuals in South Carolina found that eligible individuals who received group visits had a reduced risk of premature birth, low birthweight, and neonatal intensive care unit stays [26]. Another study suggests establishing routine care and decreasing acute care in this population [9]. Future interventions, including repeated screening throughout pregnancy and postpartum, could also assist in the early detection and management of DAP.

This study documents high rates of comorbidity within and across behavioral health conditions, including SUD, and higher use of SUD treatment among those with DAP. Given the overlap of these conditions in this and in other studies [27,28], this finding also reiterates the importance of both early detection and appropriately tailored treatment that addresses needs of those with multiple behavioral health comorbidities.

### Limitations

Diagnoses represent detected illness, yet DAP frequently goes undiagnosed, especially in non-White individuals; furthermore, race/ethnicity groups may approach treatment for DAP differently [29,30]. Systemic racism can also yield barriers for non-White individuals to accessing perinatal physical and mental health care [31,32]. This creates heterogeneity in under-diagnosis, which may skew our results. Residual bias may have also occurred such that we could not account for all possible confounding influences on the relationship between DAP and outcomes. Imperfect timing of diagnoses in claims data during healthcare visits relative to onset of illness may also have influenced study findings. Claims data can only indicate current diagnoses and may not accurately reflect symptom onset, severity of symptoms, or remission.

This study used a retrospective, observational design, which cannot establish causality. This study included individuals with continuous enrollment, yet Medicaid churn for postpartum individuals remains pervasive [33,34]. More than half of the individuals originally identified in our study population did not meet criteria for continuous enrollment. Therefore, the true extent of the impact of their DAP on healthcare utilization remains unknown and hard to accurately quantify. Individuals enrolled in Medicaid may have higher and more severe rates of DAP; findings may not necessarily generalize to non-Medicaid populations; however, individuals without continuous coverage may have higher rates of DAP than those with continuous coverage. Further, findings may not generalize to Medicaid populations in other states, as each state may handle access to care and coverage differently. For all these reasons, we anticipate that these findings represent an underestimate of the impact of DAP. We focused this study on DAP; individuals experiencing mental health conditions during pregnancy have an increased risk of maintaining or developing those conditions postpartum compared to people without DAP.

## 5. Conclusions

Accounting for sociodemographic and clinical characteristics, we found that childbearing individuals with DAP with Medicaid insurance coverage in Michigan had higher rates of adverse birth outcomes and healthcare utilization compared to those without DAP. Given the well-established negative intergenerational effects of inadequate care for childbearing individuals on outcomes for their infants, early detection and tailored, appropriate treatment of DAP may help mitigate the health burdens associated with these complex yet treatable disorders.

## Figures and Tables

**Table 1:** Demographic and clinical characteristics of deliveries with and without DAP diagnoses in Michigan Medicaid, 2012–2021.

Characteristic	DAP (N = 29,665)		No DAP (N = 189,225)		All (N = 218,890)	
	N	%	N	%	N	%
**Age at delivery**						
15–24	9429	31.78	71,892	37.99	81,321	37.15
25–39	19,557	65.93	113,734	60.11	133,291	60.89
40–44	679	2.29	3599	1.90	4278	1.95
Age at delivery, mean (SD)	27.60	5.62	26.78	5.52	26.90	5.54
**Race/ethnicity**						
American Indian/Alaskan Native/ Native Hawaiian/Pacific Islander/Asian	792	2.67	3605	1.91	4397	2.01
Black	6073	20.47	70,250	37.13	76,323	34.87
Hispanic	1614	5.44	12,330	6.52	13,944	6.37
White	20,642	69.58	95,691	50.57	116,333	53.15
None of the above	544	1.83	7349.00	3.88	7893.00	3.61
**Urbanicity**						
Urban	23,637	79.68	165,653	87.54	189,290	86.48
Non-urban	6023	20.30	23,546	12.44	29,569	13.51
Unknown	5	0.02	26	0.01	31	0.01
**Federal poverty level**						
0%	17,967	60.57	105,492	55.75	123,459	56.40
>0% to ≤50%	3555	11.98	21,535	11.38	25,090	11.46
>50%	8003	26.98	59,477	31.43	67,480	30.83
Unknown	140	0.47	2721	1.44	2861	1.31
**Delivery-related outcomes**						
Cesarean delivery	10,525	35.48	59,468	31.43	69,993	31.98
Preterm delivery	4933	16.63	26,760	14.14	31,693	14.48
30-day readmission after delivery	793	2.67	3950	2.09	4743	2.17
SMM within 42 days	1163	3.92	6104	3.23	7267	3.32
**Clinical characteristics**						
Bateman OBCMI score, mean (SD) ^†^	2.07	2.18	1.27	1.76	1.38	1.85
**Behavioral health diagnoses**						
Any diagnosis ^‡^	14,699	49.55	18,173	9.60	32,872	15.02
Serious mental illness	3773	12.72	3625	1.92	7398	3.38
Substance use disorder	6265	21.12	8346	4.41	14,611	6.68
Other behavioral health diagnosis	9877	33.30	9339	4.94	19,216	8.78
**Prescription medication use**						
Any psychotropic medication	21,266	71.69	23,603	12.47	44,869	20.50
Antidepressants	18,450	62.19	17,879	9.45	36,329	16.60
Anxiolytics	7557	25.47	5818	3.07	13,375	6.11
Antipsychotics	4044	13.63	2944	1.56	6988	3.19
Mood stabilizers	182	0.61	173	0.09	355	0.16
Stimulants	2139	7.21	2214	1.17	4353	1.99
Opioid analgesics	16,507	55.64	86,779	45.86	103,286	47.19
Medication for opioid use disorder	1960	6.61	1876	0.99	3836	1.75

^‡^ Coded based on delivery hospitalization; ^†^ Excluding depression and anxiety. Abbreviations: depression and/or anxiety during pregnancy (DAP), standard deviation (SD), obstetric comorbidity index (OBCMI). Other behavioral health diagnosis includes adjustment disorders, attention-deficit, conduct, and disruptive behavior disorders, bipolar disorder, miscellaneous mental health disorders, personality disorders, post-traumatic stress disorder (PTSD), other mood disorders, other psychotic disorders, schizophrenia, suicide and intentional self-inflicted injury, suicidal ideation.

**Table 2:** Unadjusted and adjusted odds ratios of delivery-related outcomes and utilization of health services among deliveries with and without DAP in Michigan Medicaid, 2012–2021.

	Unadjusted		Adjusted *	
Delivery-related outcomes	OR	95% CI	OR	95% CI
Caesarean delivery	1.15	(1.12, 1.18)	1.02	(1.00, 1.05)
Preterm delivery	1.21	(1.16, 1.25)	1.15	(1.11, 1.19)
30-day readmission after delivery	1.21	(1.14, 1.29)	1.14	(1.07, 1.22)
SMM within 42 days	1.29	(1.19, 1.39)	1.27	(1.18, 1.38)
**Utilization of health services**				
Ambulatory care visits	8.32	(7.26, 9.53)	7.75	(6.75, 8.88)
Inpatient visits	1.12	(1.10, 1.14)	1.13	(1.11, 1.15)
ED/observation visits	1.72	(1.67, 1.77)	1.86	(1.80, 1.92)
Psychotherapy visits	180.85	(168.14, 194.51)	172.83	(160.10, 186.58)
SUD visits	7.17	(6.89, 7.46)	5.61	(5.37, 5.85)

* Adjusted for delivery age, race/ethnicity, urbanicity, federal poverty level, obstetric comorbidities. Abbreviations: Depression and/or anxiety during pregnancy (DAP), odds ratio (OR), confidence interval (CI).

## Data Availability

The data that support the findings of this study are available from the MDHHS Data Warehouse. Restrictions apply to the availability of these data, which were used under license for this study and thus are not publicly available. Data from the MDHHS Data Warehouse for this study was made available through a Data Use Agreement with MDHHS by program sponsors Michigan Pregnancy Risk Assessment Monitoring System, Medical Services Administration, Behavioral Health and Developmental Disabilities Administration, and Vital Records.

## Appendix A. Cohort and Variable Definitions

*Delivery Codes*

Variable	ICD Diagnosis Code	ICD Procedure Code	DRG	CPT/HCPCS
Cesarean	-	740, 741, 742, 744, 7499; 10D00Z0, 10D00Z1, 10D00Z2	370, 371, 765, 766, 540, 5401, 5402, 5403, 5404	59510, 59514, 59515, 59618, 59620, 59622
Vaginal		10D07Z3, 10D07Z4, 10D07Z5, 10D07Z6, 10D07Z7, 10D07Z8, 10E0XZZ	372, 373, 374, 375, 767 768, 774, 775, 541, 542, 560, 5411, 5412, 5413, 5414, 5421, 5422, 5423, 5424, 5601, 5602, 5603	59400, 59409, 59410, 59610, 59612, 59614
Preterm Birth	O601, O6010, O6010X0, O6010X1, O6010X2, O6010X3, O6010X4, O6010X5, O6010X9, O6012, O6012X0, O6012X1, O6012X2, O6012X3, O6012X4, O6012X5, O6012X9, O6013, O6013X0, O6013X1, O6013X2, O6013X3, O6013X4, O6013X5, O6013X9, O6014, O6014X0, O6014X1, O6014X2, O6014X3, O6014X4, O6014X5, O6014X9			

*Mental Health Disorder Codes*

Variable		ICD Diagnosis Code
Depression and/or Anxiety (DAP)	Anxiety	300.xx, 308.xx, 313.xx, 293.xx, F06.xx, F40.xx, F41.xx, F42.xx, F43.xx, F48.xx, R45.xx
	Depression	311.xx, 296.xx, 300.xx, F32.xx, F33.xx
Other Behavioral Health Conditions	Mental Health Disorders, excluding DAP	312.xx, 314.xx, 313.xx, F90.xx, F91.xx, R46.xx, R46.xx, R46.xx, R46.xx, 309.xx, 309.xx, F43.xx

*Psychotropic Medication Classification Codes*

Medication class	HIC 3 Specific Therapeutic Code
Antidepressant	H24, H2H, H2J, H2K, H2N, H2S, H2U, H2Y, H7B, H7C, H7D, H7E, H7I, H7J, H7K, H7L, H8P, H8S, H8T, H8Z
Antidepressant/antipsychotic	H2W, H7A
Antidepressant/anxiolytic	H2X, H7M
Antipsychotic	H2G, H2I, H2L, H2O, H7O, H7P, H7Q, H7R, H7S, H7T, H7U, H7V, H7X, H7Z, H8N, H8W
Anxiolytic	H20, H2F, H2P, H8A, H8K
Mood stabilizer	H2M
Opioid/narcotic analgesic	H30, H3A, H3B, H3H, H3J, H3M, H3N, H3Q, H3R, H3U, H3X, H3Z, S7G
Opioid/narcotic antagonist	H3T, H3W, H3Y, H33
Opioid/narcotic antitussive	B4F, B4N, B6C, B6I, B6U
Stimulant	H2V, H7Y, H8M, H8Q, J5B

*Visit Codes*

Visit Type	ICD Diagnosis Code	ICD Procedure Code	CPT/HCPCS	Revenue	Bill Type
Ambulatory Care	Z00.00, Z00.01, Z00.121, Z00.129, Z00.3, Z00.5, Z00.8, Z02.0, Z02.1, Z02.2, Z02.3, Z02.4, Z02.5, Z02.6, Z02.71, Z02.79, Z02.81, Z02.82, Z02.83, Z02.89, Z02.9, Z76.1, Z76.2, V20.2, V70.0, V70.3, V70.5, V70.6, V70.8, V70.9		92002, 92004, 92012, 92014, 99201, 99202, 99203, 99204, 99205, 99211, 99212, 99213, 99214, 99215, 99241, 99242, 99243, 99244, 99245, 99304, 99305, 99306, 99307, 99308, 99309, 99310, 99315, 99316, 99318, 99324, 99325, 99326, 99327, 99328, 99334, 99335, 99336, 99337, 99341, 99342, 99343, 99344, 99345, 99347, 99348, 99349, 99350, 99381, 99382, 99383, 99384, 99385, 99386, 99387, 99391, 99392, 99393, 99394, 99395, 99396, 99397, 99401, 99402, 99403, 99404, 99411, 99412, 99429, 99461, 99483, G0463, T1015, G0402, G0438, G0439, S0620, S0621, 98966, 98967, 98968, 99441, 99442, 99443	510, 511, 512, 513, 514, 515, 516, 517, 519, 520, 521, 522, 523, 524, 525, 526, 527, 528, 529, 982, 983	
Inpatient				100, 1000, 1001, 1002, 1003, 1004, 1005, 101, 110, 111, 112, 113, 114, 116, 117, 118, 119, 120, 121, 122, 123, 124, 126, 127, 128, 129, 130, 131, 132, 133, 134, 136, 137, 138, 139, 140, 141, 142, 143, 144, 146, 147, 148, 149, 150, 151, 152, 153, 154, 156, 157, 158, 159, 160, 164, 167, 169, 170, 171, 172, 173, 174, 179, 190, 191, 192, 193, 194, 199, 200, 201, 202, 203, 204, 206, 207, 208, 209, 210, 211, 212, 213, 214, 219, 22, 24, 524, 525, 550, 551, 552, 559, 660, 661, 662, 663, 669, 720, 721, 722, 723, 724, 729, 987	180, 181, 182, 183, 184, 185, 187, 188, 210, 211, 212, 213, 214, 215, 217, 218, 220, 221, 222, 223, 224, 225, 227, 228, 280, 281, 282, 283, 284, 285, 287, 288, 289, 650, 652, 653, 654, 655, 657, 658, 660, 662, 663, 664, 665, 667, 668, 860, 862, 863, 864, 865, 867, 868, 018F, 018G,018H, 018I, 018J, 018K, 018M, 018O, 018X, 018Y, 018Z, 021F, 021G, 021H, 021I, 021J, 021K, 021M, 021O, 021X, 021Y, 021Z, 022F, 022G, 022H, 022I, 022J, 022K, 022M, 022O, 022X, 022Y, 022Z, 028F, 028G, 028H, 028I, 028J, 028K, 028M, 028O, 028X, 028Y, 028Z, 065F, 065G, 065H, 065I, 065J, 065K, 065M, 065O, 065X, 065Y, 065Z, 066F, 066G, 066H, 066I, 066J, 066K, 066M, 066O, 066X, 066Y, 066Z, 086F, 086G, 086H, 086I, 086J, 086K, 086M, 086O, 086X, 086Y, 086Z
Emergency Department and/or observation			99281, 99282, 99283, 99284, 99285, G0380, G0381, G0382, G0383, G0384 EDsurgprocPOS23 if place of service = 23 AND CPT between 10050–69979 ObsProc if CPT codes: 99217, 99218, 99219, 99220, G0378, G0379 ObsRev if revenue code 0762	045, 0981	
Psychotherapy			90804, 90805, 90806, 90807, 90808, 90809, 90810, 90811, 90812, 90813, 90814, 90815, 90816, 90817, 90818, 90819, 90820, 90821, 90822, 90823, 90824, 90826, 90827, 90828, 90829, 90832, 90833, 90834, 90836, 90837, 90838, 90839, 90840, 90841, 90842, 90843, 90844, 90846, 90847, 90849, 90853, 90855		
Substance Use Disorder Related	291.xx, 357.xx, 425.xx, 535.xx, 571.xx, 980.xx, 303.xx, 305.xx, 760.xx, F10.xx, G62.xx, I42.xx, K29.xx, K70.xx, O99.xx, 304.xx, F12.xx, F14.xx, 292.xx, 779.xx, 648.xx, 655.xx, 965.xx, F55.xx, O35.xx, F16.xx, F18.xxP04.xx, P96.xx, Q86.xx, F11.xx, F15.xx, F19.xx, F13.xx, T36.xx, T37.xx, T38.xx, T39.xx, T40.xx, T41.xx, T42.xx, T43.xx, T44.xx, T45.xx, T46.xx, T47.xx, T48.xx, T49.xx, T50.xx, T51.xx, T52.xx, T53.xx, T54xx, T55.xx, T56.xx, T57.xx, T58.xx, T59.xx, T60.xx, T61.xx, T62.xx, T63.xx, T64.xx, T65.xx, T71.xx, V65.xx, F17.xx	9462, 9461, 9463, 946, 9446, 9469, 9468, 9467, 9465, 9464, 9466	G0397, G0396, H0048, H2034, H0014, H0027, H0029, H0007, H0016, H0026, H0028, H0003, H0009, H0005, H0015, H001, H0012, H0011, H0013, H0020, H0008, T1006, H0050, H0022, H0006, H0021, H0047, H2036, H2035, H0001, H0049, T102, T1007, T1011, S9475, G0442, H0039, H0040, H0004, H2012, H0030, H0023, H0025, H0017, H0024, H0002, H0019, H018, G0443, T1009, H2015, H2016, H2011, T1008, T1010, H2025, H2026, H0033, H2001, H0038, H2014, H2019, H2020, H2ZZZZ, HZ63ZZZ, HZ33ZZZ, GZ3ZZZZ, HZ82ZZZ, HZ83ZZZ, HZ87ZZZ, HZ86ZZZ, HZ81ZZZ, HZ85ZZZ, HZ84ZZZ, HZ80ZZZ, HZ89ZZZ, HZ88ZZZ, HZ93ZZZ, HZ97ZZZ, HZ96ZZZ, HZ92ZZZ, HZ95ZZZ, HZ94ZZZ, HZ98ZZZ, HZ91ZZZ, HZ90ZZZ, HZ99ZZ		

*Severe Maternal Morbidity Codes*

Variable	ICD Diagnosis Code	ICD Procedure Code
Acute Myocardial Infarction	410.xx, I21.01, I21.02, I21.09, I21.11, I21.19, I21.21, I21.29, I21.3, I21.4, I21.9, I21.A1 and I21.A9, I22.0, I22.1, I22.2, I22.8, I22.9	
Aneurysm	441.xx, I71.00–I71.03, I71.1, I71.2, I71.3, I71.4, I71.5, I71.6, I71.8, I71.9, I79.0	
Acute Renal Failure	584.5, 584.6, 584.7, 584.8, 584.9, 669.3x, N17.0, N17.1, N17.2, N17.8, N17.9, O90.4	
Adult Respiratory Distress Syndrome	518.5x, 518.81 518.82 518.84, 799.1, J80, J95.1, J95.2, J95.3, J95.821, J95.822, J96.00, J96.01, J96.02, J96.20, J96.21, J96.22, R09.2	
Amniotic Fluid Embolism	673.1x, O88.11x *, O88.12, O88.13 * x = 1st, 2nd, and 3rd trimester	
Cardiac Arrest/Ventricular Fibrillation	427.41, 427.42, 427.5, I46.2, I46.8, I46.9, I49.01, I49.02	
Conversion of Cardiac Rhythm		99.6x, 5A2204Z, 5A12012
Disseminated Intravascular Coagulation	286.6, 286.9, 666.3x, D65, D68.8, D68.9, O72.3	
Eclampsia	642.6x, O15.00, O15.02, O15.03, O15.1, O15.2, O15.9, O14.22, O14	
Heart Failure/Arrest during Surgery or Procedure	997.1, I97.120, I97.121, I97.130, I97.131, I97.710, I97.711	
Puerperal Cerebrovascular Disorders	430.xx, 431.xx, 432.xx, 433.xx, 434.xx, 436xx, 437.xx, 671.5x, 674.0x, 997.02, I60.0x, I60.1x, I60.2, I60.3x, I60.4, I60.5x, I60.6, I60.7, I60.8, I60.9; I61.1, I61.2, I61.3, I61.4, I61.5, I61.6, I61.8, I61.9; I62.0x, I62.1, I62.9; I63.0xx, I63.1xx, I63.2xx, I63.3xx, I63.4xx, I63.5xx, I63.6, I63.8, I63.9; I65.0x, I65.1, I65.2x, I65.8, I65.9; I66.0x, I66.1x, I66.2x, I66.3, I66.8, I66.9; I67.0, I67.1, I67.2, I67.3, I67.4, I67.5, I67.6, I67.7, I67.8xx, I67.9; I68.0, I68.2, I68.8; O22.51, O22.52, O22.53, I97.810, I97.811, I97.820, I97.821, O87.3, 674.0x	
Pulmonary edema/Acute Heart Failure	518.4, 428.1, 428.0, 428.21, 428.23, 428.31, 428.33, 428.41, 428.43, J81.0, I50.1, I50.20, I50.21, I50.23, I50.30, I50.31, I50.33, I50.40, I50.41, I50.43, I50.9	
Severe Anesthesia Complications	668.0x, 668.1x, 668.2x, O74.0, O74.1, O74.2, O74.3, O89.01, O89.09, O89.1, O89.2	
Sepsis	038.xx, 995.91, 995.92, 670.2x (after October 1, 2009); O85, O86.04, T80.211A, T81.4XXA, T81.44, T81.44XA, T81.44XD, T81.44XS, R65.20 or A40.0, A40.1, A40.3, A40.8, A40.9, A41.01, A41.02, A41.1, A41.2, A41.3, A41.4, A41.50, A41.51, A41.52, A41.53, A41.59, A41.81, A41.89, A41.9, A32.7	
Shock	669.1x, 785.5x, 995.0, 995.4, 998.0x, O75.1, R57.0, R57.1, R57.8, R57.9, R65.21, T78.2XXA, T88.2XXA, T88.6XXA, T81.10XA, T81.11XA, T81.19XA	
Sickle Cell Disease with Crisis	282.42, 282.62, 282.64, 282.69, D57.00, D57.01, D57.02, D57.211, D57.212, D57.219, D57.411, D57.412, D57.419, D57.811, D57.812, D57.819 (5th digit: unspecified, acute chest syndrome or splenic sequestration)	
Air and Thrombotic Embolism	415.1x, 673.0x, 673.2x 673.3x, 673.8x, I26.01, I26.02, I26.09, I26.90, I26.92, I26.99, O88.011-O88.019, 088.02, O88.03, O88.211-O88.219, O88.22, O88.23, O88.311-O88.319, O88.32, O88.33, O88.81, O88.82, O88.83	
Blood Products Transfusion		99.0x, 30233H1, 30233L1, 30233K1, 30233M1, 30233N1, 30233P1, 30233R1, 30233T1, 30233H0, 30233L0, 30233K0, 30233M0, 0233N0, 30233P0, 30233R0, 30233T0, 30230H1, 30230L1, 30230K1, 30230M1, 30230N1, 30230P1, 30230R1, 30230T1, 30230H0, 30230L0, 30230K0, 30230M0, 30230N0, 30230P0, 30230R0, 30230T0, 30240H1, 30240L1, 30240K1, 30240M1, 30240N1, 30240P1, 30240R1, 30240T1, 30240H0, 30240L0, 30240K0, 30240M0, 30240N0, 30240P0, 30240R0, 30240T0, 30243H1, 30243L1, 30243K1, 30243M1, 30243N1, 30243P1, 30243R1, 30243T1, 30243H0, 30243L0, 30243K0, 30243M0, 30243N0, 30243P0, 30243R0, 30243T0, 30250H1, 30250L1, 30250K1, 30250M1, 30250N1, 30250P1, 30250R1, 30250T1, 30250H0, 30250L0, 30250K0, 30250M0, 30250N0, 30250P0, 30250R0, 30250T0, 30253H1, 30253L1, 30253K1, 30253M1, 30253N1, 30253P1, 30253R1, 30253T1, 30253H0, 30253L0, 30253K0, 30253M0, 30253N0, 30253P0, 30253R0, 30253T0, 30260H1, 30260L1, 30260K1, 30260M1, 30260N1, 30260P1, 30260R1, 30260T1, 30260H0, 30260L0, 30260K0, 30260M0, 30260N0, 30260P0, 30260R0, 30260T0, 30263H1, 30263L1, 30263K1, 30263M1, 30263N1, 30263P1, 30263R1, 30263T1, 30263H0, 30263L0, 30263K0, 30263M0, 30263N0, 30263P0, 30263R0, 30263T0
Hysterectomy		68.3x-68.9x, 0UT90ZZ, 0UT94ZZ, 0UT97ZZ, 0UT98ZZ, 0UT9FZZ
Temporary Tracheostomy		31.1, 0B110Z4, 0B110F4, 0B113Z4, 0B113F4, 0B114Z4, 0B114F4
Ventilation		93.90, 96.01, 96.02, 96.03, 96.05, 5A1935Z, 5A1945Z, 5A1955Z

*Bateman Obstetric Comorbidity Index*

Variable	ICD Diagnosis Code
Bateman Obstetric Comorbidity Index	291, 303, 3050, 493, 394, 395, 396, 397, 424, 42822, 42823, 42832, 42833, 42842, 42843, 412, 413, 414, 581, 582, 583, 585, 587, 588, 6462, 7450, 7451, 7452, 7453, 7454, 7455, 7456, 7457, 7458, 7459, 7460, 7461, 7462, 7463, 7464, 7465, 7466, 7467, 7468, 7469, 7470, 7471, 7472, 7473, 7474, 6485, 304, 3052, 3053, 3054, 3055, 3056, 3057, 3058, 3059, 6483, 6423, 042, V08, 6424, 6427, 651, V272, V273, V274, V275, V276, V277, V278, 6410, 6411, 250, 6480, 401, 402, 403, 404, 405, 6420, 6421, 6422, 6427, 6542, 4160, 4168, 4169, 6425, 6466, 2824, 2826, 7100, F10, J44, J45, I05, I06, I07, I08, I09, I34, I35, I36, I37, I38, I39, I500, I20, I25, N022, N03, N04, N05, N08, N171, N172, N18, N25, O268, Q20, Q21, Q22, Q23, Q24, Q25, Q26, O994, F11, F12, F13, F14, F15, F16, F18, F19, O13, O16, B20, B24, O987, Z21, O11, O14, O30, O31, Z372, Z373, Z374, Z375, Z376, Z377, Z3790, O44, E10, E11, O245, O246, O247, I10, I11, I12, I13, I15, O10, O11, O3420, I270, I272, I278, I279, O14, O15, D56, D57, M32
